# Targeting Mitochondrial OXPHOS and Their Regulatory Signals in Prostate Cancers

**DOI:** 10.3390/ijms222413435

**Published:** 2021-12-14

**Authors:** Chia-Lin Chen, Ching-Yu Lin, Hsing-Jien Kung

**Affiliations:** 1 Ph.D. Program for Cancer Biology and Drug Discovery, College of Medical Science and Technology, Taipei Medical University, Taipei 110, Taiwan; truip75@gmail.com (C.-L.C.); cylin071@tmu.edu.tw (C.-Y.L.); 2 Research Center of Cancer Translational Medicine, Taipei Medical University, Taipei 110, Taiwan; 3 Institute of Molecular and Genomic Medicine, National Health Research Institutes, Zhunan, Miaoli County 350, Taiwan; 4 Comprehensive Cancer Center, Department of Biochemistry and Molecular Medicine, University of California at Davis, Sacramento, CA 95817, USA

**Keywords:** mitochondria, OXPHOS, cancer therapy

## Abstract

Increasing evidence suggests that tumor development requires not only oncogene/tumor suppressor mutations to drive the growth, survival, and metastasis but also metabolic adaptations to meet the increasing energy demand for rapid cellular expansion and to cope with the often nutritional and oxygen-deprived microenvironment. One well-recognized strategy is to shift the metabolic flow from oxidative phosphorylation (OXPHOS) or respiration in mitochondria to glycolysis or fermentation in cytosol, known as Warburg effects. However, not all cancer cells follow this paradigm. In the development of prostate cancer, OXPHOS actually increases as compared to normal prostate tissue. This is because normal prostate epithelial cells divert citrate in mitochondria for the TCA cycle to the cytosol for secretion into seminal fluid. The sustained level of OXPHOS in primary tumors persists in progression to an advanced stage. As such, targeting OXPHOS and mitochondrial activities in general present therapeutic opportunities. In this review, we summarize the recent findings of the key regulators of the OXPHOS pathway in prostate cancer, ranging from transcriptional regulation, metabolic regulation to genetic regulation. Moreover, we provided a comprehensive update of the current status of OXPHOS inhibitors for prostate cancer therapy. A challenge of developing OXPHOS inhibitors is to selectively target cancer mitochondria and spare normal counterparts, which is also discussed.

## 1. Introduction

Prostate cancer (PCa) represents one of the most frequently diagnosed malignancies among men worldwide. Based on the survey of Global Cancer Observatory in 2020, the incidence of PCa ranks as the top two cancers for men and top one cancer for men with age over 50 (http://gco.iarc.fr/ accessed on 1 October 2021). As society ages, PCa has become an increasingly important medical issue. At the early stage, PCa is often treatable by hormone therapy; however, the survival rate drops dramatically if the tumor becomes resistant to anti-androgen therapies, which are often referred to as castration-resistant prostate cancer (CRPC) cells. Ninety percent of patients receiving hormone therapy will finally relapse and become hormone-refractory within 2–3 years [1]. Currently, there is no effective treatment for the relapsed PCa. Therefore, there is an urgent need to identify intervention targets and develop therapeutics to overcome castration resistance.

Warburg effect, the metabolic switch from oxidative phosphorylation (OXPHOS) to aerobic glycolysis, has been observed in a variety of tumor types [2]. While the diminished function of mitochondria relative to glycolysis seems to be a hallmark of Warburg and Warburg originally proposed that defects of mitochondria cause cancer, we now know that mitochondrial functions are still required, and in metastatic cancers with EMT properties, OXPHOS functions are important to sustain the required energy for migration and invasion. Warburg effect’s true definition also includes the preferential increase in cancer cells of the glucose uptake and the production of lactate in the presence of oxygen. The increased production of glucose in the presence of oxygen itself would also increase mitochondrial functions. Thus, the increase in glycolysis does not necessarily exclude mitochondrial activity, and that itself does not “predict the loss of mitochondrial functions” [3].

**Figure 1:**
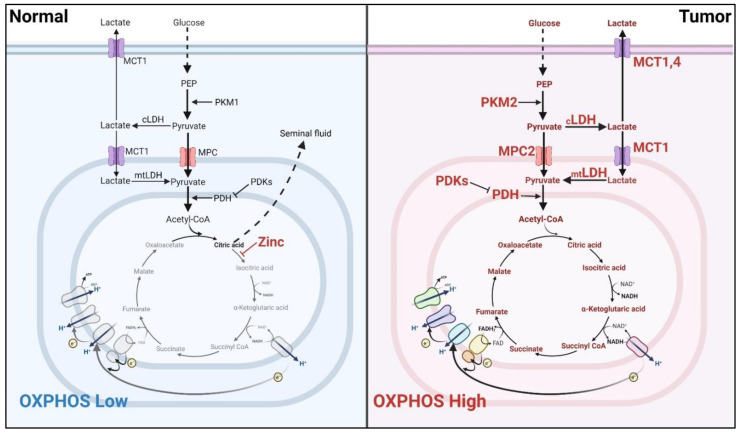
Metabolic switch during prostate tumorigenesis. In normal prostate cells, zinc ion impedes the TCA cycle to release the citrate for seminal fluid production, resulting in the low level of mitochondrial respiration activity (**left panel**). During the prostate tumor progression, Pca cells overexpress the glycolytic enzymes (including PKM2, MPC2, MCT, mtLDH, PDH, PDKs) to drive the pyruvate flux into the mitochondrial TCA cycle, leading to the high level of mitochondrial respiration activity (**right panel**).

Increased glycolysis provides an opportunity for cancer cells to grow in a hypoxic environment and the diminished mitochondrial function lessens free radical damage and apoptosis caused by mitochondria-mediated ROS [4]. However, this may not be the case for the PCa (Figure 1). In normal prostatic tissue, prostate epithelial cells employ a short circuit of the citric acid cycle to sustain physiological citrate secretion [5]. Zinc ion inhibits the mitochondrial aconitase (ACO_2_) activity to prevent citrate from entering the next step, which leads to the disruption of the tricarboxylic acid (TCA) cycle. Hence, the normal prostate cells exhibit heightened glycolysis relative to OXPHOS as compared to other tissues [6].

During the early development of PCa, the concentration of zinc declines, which allows PCa cells to consume the citrate to power OXPHOS [7] or fuel lipogenesis [8]. By contrast, the elevated activity of glycolysis seems to return at a later stage of PCa progression and is associated with castration assistance [9]. Clinically, ^18^F-FDG-PET/CT imaging shows no significant changes in glucose uptake in most primary PCa [10]. Moreover, hyperpolarized [1-^13^C] pyruvate MRI shows an elevated flux of [1-^13^C] pyruvate to [1-^13^C] lactate in high-grade and metastatic tissue [11,12]. These clinical data confirm this unique metabolic switch during the PCa progression.

In this review, we will discuss the key regulators of metabolic adaptations of PCa progression and discuss the contrasting views of literature. Finally, we will summarize the recent progress of the OXPHOS-targeting strategy for PCa therapy.

## 2. OXPHOS in PCa

Aberrant OXPHOS gene expression in PCa was first observed by Pardee’s group in 1996 [13]. One of the cytochrome c oxidase subunits, COXVIc, was highly expressed in tumor samples and in PCa cell lines as well. In 2013, Grupp et al. evaluated MCT02, a marker for mitochondrial content, in 11,152 PCa specimens and found the expression of MCT02 is associated with clinical progression indices, including tumor stage, Gleason grade, and lymph node metastasis (all *p*-value < 0.0001) [14]. Later, Kelly et al. profiled seven metabolic pathways in 404 patients and found that the OXPHOS pathway is significantly upregulated in malignant tumors [15]. Reznik et al. analyzed the metabolites in paired samples and found decreased levels of citrate and lactate in PCa [16]. These studies indicate an important role of mitochondrial OXPHOS in PCa progression.

## 3. Transcriptional Regulation of OXPHOS in PCa

**Figure 2:**
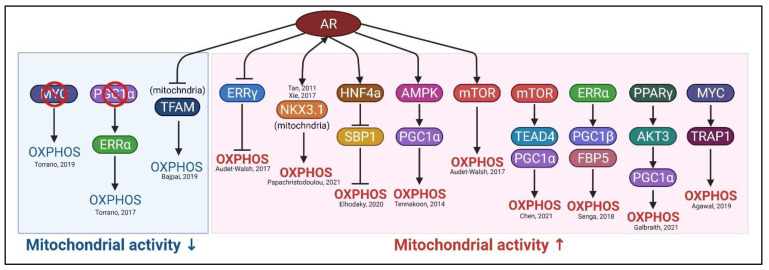
Summary of transcriptional regulations of OXPHOS pathway in PCa. In PCa, the OXPHOS pathway can be regulated by various transcription factors that lead to increased mitochondrial activity (red) or decreased mitochondrial activity (blue).

OXPHOS is conducted in mitochondria by five enzymatic complexes, consisting of 88 proteins, 75 of which are nuclear encoded, and 13 mitochondrial encoded [17]. The activities of OXPHOS are regulated by the expression levels, the assembly of the complexes, and the intactness of these genes. It is also influenced by the metabolic flow (e.g., pyruvate, succinate) of the TCA cycle. In this section, we will discuss the regulation of the expression of the nuclear-encoded OXPHOS genes, with attention to transcription factors associated with PCa progression (Figure 2).

**AR:** As a driver of PCa progression, androgen receptor (AR) plays a vital role not only in gene regulation [18] but also in metabolic rewiring [19,20]. Audet-Walsh et al. showed that AR regulates glycolysis, mitochondrial OXPHOs, and lipid synthesis by suppression of estrogen-related receptor γ (ERRγ) [21]. They further showed that AR stimulates the mTOR pathway and directly interacts with nuclear mTOR to upregulate the OXPHOS gene expression [22]. AR can also activate the OXPHOS pathway by inducing HNF4a expression, which suppresses the expression of SBP1 (selenium-binding protein 1), a negative regulator of OXPHOS [23]. On the other hand, Bajpai et al. showed that a fraction of AR is able to translocate to mitochondria where it negatively regulates TFAM and the expression of OXPHOS assembly factors as well as the stability of OXPHOS super-complex [24]. These results suggest AR, depending on its cellular localization, is able to modulate the OXPHOS pathway via interacting with various co-factors.

**NKX3.1:** NK3 homeobox 1, a prostate-specific transcription factor, plays a key role in both prostate development [25] as well as tumor progression at different stages [26]. It has been shown that the expression of NKX3.1 can be regulated by AR [27], and NKX3.1 also can regulate the AR transcriptional network by forming a transcriptional complex of AR/NKX3.1/FoxA1 [28]. Recently, Papachristodoulou et al. showed that NKX3.1 can translocate into mitochondria by a chaperone protein HSPA9 and regulate the expression of mitochondrial-encoded ETC genes and OXPHOS functions [29].

**ERR:** The estrogen-related receptors (ERRs), another family of nuclear steroid hormone receptors, also play key roles in metabolic regulation in PCa [30]. Among the three ERR isoforms (α, β, and γ), ERRα is overexpressed whereas the other two isoforms are suppressed in PCa [31,32]. As already described, ERRγ and ERRα are both involved in the regulation of mitochondrial activities in PCa cells. PGC-1a forms a transcriptional complex with ERRα to mediate the transcription of nuclear-encoded mitochondrial genes [33]. Similarly, PGC1β and FABP5 (fatty acid-binding protein 5) are associated with ERRα to activate downstream metabolic genes, such as ATP5B involved in OXPHOS [34].

**MYC:** Myc is well recognized for its oncogenic role in PCa progression and as a modulator of tumor metabolism [35]. Based on chromosomal immunoprecipitation-sequencing (ChIP-seq) analysis, four hundred nuclear-encoded mitochondrial genes were identified as MYC targets, including the genes associated with OXPHOS complexes, mitochondrial transcription/translation factors, mitochondrial ribosomes, protein transporters, and other transcription factors involved in mitochondrial biogenesis [36]. In PCa, MYC not only plays a key role in regulating mitochondrial dynamics but also targets the mitochondrial chaperone, TRAP1, to mediate the mitochondrial protein folding and function of OXPHOS [37].

**TEAD4**: Our recent finding identified a member of the TEA domain transcription factor family, TEAD4, which is overexpressed in PCa, modulates OXPHOS gene expression and mitochondrial function [38]. We showed that in PCa, activation of mTOR pathway by arginine induces TEAD4 nuclear translocation to OXPHOS promoter region where it forms a complex with PGC-1α and together with histone acetylases coordinately upregulate nuclear-encoded OXPHOS gene expression. Conversely, silencing of TEAD4 reduces mitochondrial respiration activity, resulting in mtROS production, mitochondrial dysfunction, and PCa cell death.

**PGC-1α:** Another master regulator of mitochondrial OXPHOS, peroxisome proliferator-activated receptor gamma coactivator 1 (PGC1α), is highly expressed in PCa cell lines [39]. Shiota et al. further showed that PGC1α directly interacts with AR and activates AR transcriptional activity [39]. Later, Tennakoon et al. showed that AR signaling induces PGC1α via the AMPK pathway, and this AR-induced AMPK-PGC1α axis controls the metabolic activities of PCa [40]. Galbraith et al. showed that PPARγ induced AKT3 expression to promote PGC1α nuclear translocation and mitochondrial biogenesis in PCa [41]. By contrast, Torrano et al. showed that the PGC1α-ERRα axis is suppressed in PCa, and this axis is potentially tumor suppressive [42] and downmodulates Myc [43]. In their studies, overexpression of PGC1α in PCa cell lines with undetectable PGC1α induces OXPHOS, resulting in the reversal of the Warburg effects. Thus, the oncogenic or tumor-suppressing role of PGC1α and the effects of OXPHOS induction are highly context-dependent. Too high or too low a level of OXPHOS activities would all lead to the demise of PCa cells.

## 4. Metabolic Regulations of OXPHOS

TCA cycle is initiated by the influx of cytosolic pyruvate into mitochondria, which impacts the downstream OXPHOS reactions. Thus, the abundance of pyruvate, the efficiency of its influx into mitochondria, and conversion to acetyl-CoA are nodes of regulation. Below, we describe the regulatory circuit of pyruvate in PCa.

**PKM2**: Pyruvate kinase muscle isozyme 2 (PKM2), the key enzyme of the final and rate-limiting step for glycolysis, is generally overexpressed in high-grade malignant cancer cells [44]. PKM2 converts phosphoenolpyruvate (PEP) to pyruvate with the production of ATP. The pyruvate generated can be converted to lactate (glycolysis) or flow into mitochondria to turn on the TCA cycle and accelerate the OXPHOS reactions. A high level of pyruvate is thought to favor mitochondrial OXPHOS reaction, whereas a moderate level contributes to glycolysis. Compared to its alternately spliced sibling PKM1, which has a high pyruvate kinase activity and is abundantly expressed in normal tissues, the tumor-enriched PKM2 has a moderate pyruvate kinase activity. This was suggested to be one reason to account for the Warburg effect in tumors. In the cytosol, PKM2 tetramer is the active form, and its conversion to dimer further diminishes the pyruvate kinase activity. The enzymatic activity of PKM2 can thus be regulated by agonists (e.g., FBP, serine, SAICAR) and antagonists (e.g., alanine, phenylalanine, proline), which affects tetramer formation [45]. Interestingly, when PKM2 is converted into dimer form, a fraction of which enters the nucleus where it serves as a coactivator of HIF-1a to activate the transcription of glycolytic enzymes (HK, LDHA, etc.) and PDKs, enzymes that inhibit the conversion of pyruvate to acetyl-CoA [45] (Figure 1). As such, it further increases the flow of pyruvate to lactate and blocks the flow to mitochondria to enhance the Warburg effect. It was reported that KDM8, a histone demethylase that is overexpressed in PCa, interacts with PKM2 and facilitates dimer formation and translocation into the nucleus to enhance HIF-1a-mediated transcription of glycolytic enzymes [46,47]. At the same time, KDM8 directly interacts with AR to modulate genes associated with therapy resistance [47]. Blocking complex I activity by metformin abolishes PKM2 nuclear translocation, leading to the incased level of glucose consumption and the metabolic reprogramming to a Warburg phenotype [48]. A recent study shows that PKM2 induces the expression of the OXPHOS complex IV subunit, COXII, by interacting with ERK and promoting the c-JUN binding activity on the gene promoter region [49]. Silencing PKM2 leads to autophagic cell death via suppressing AKT/mTOR pathway in PCa cells [50]. In hypoxic conditions, PKM2 is induced and causes resistance to mTOR inhibitors [51]. These results suggest that PKM2 could be a key factor for the balance between glycolysis and OXPHOS in PCa cells. Disruption of mitochondrial homeostasis by targeting PKM2 could lead to PCa cell death.

**MPC:** Pyruvate is transported into mitochondria by a mitochondrial pyruvate carrier (MPC). MPC is a hetero-oligomeric complex of MPC1 and MPC2. This carrier transports the pyruvate into the mitochondrial matrix, which is then converted to acetyl-CoA by pyruvate dehydrogenase (PDH) for the generation of citrate to enter the TCA cycle. The expression of MPC1 and MPC2 are independently associated with clinical outcomes in PCa [52]. Bader et al. showed that in AR-driven PCa, MPC2 is highly expressed in primary tumors as well as in castration-resistant specimens [53]. The MPC2 expression is also positively correlated with AR expression in PCa cell lines. AR was found to bind to the MPC2 locus and direct the transcription of MPC2. In vitro inhibition of MPC2 alters mitochondrial functions and morphology. In vivo inhibition of MPC significantly reduced tumor growth. Lee et al. further showed that androgen induces the expression of mitochondrial fission mediator, DRP1, as well as MPC2 expression, leading to increased mitochondrial function [54]. These studies illustrated that AR upregulates MPC to import pyruvate to fuel OXPHOS, lipogenesis, and other biosynthetic processes necessary for PCa growth.

By contrast, Wang et al. reported that MPC1 is down-regulated and associated with prostate tumor progression [55]. By searching the MPC1 promoter region, they identified a steroid receptor superfamily member, COUP-TFII. The expression of COUP-TFII is negatively correlated with MPC1 in prostate tumor specimens. Silencing of COUP-TFII increases the MPC1 expression, which leads to induction of lactate production, glucose consumption, and glycolytic activity. Intriguingly, silencing of MPC1 diminishes the effect of COUP-TFII on glycolysis in vitro and tumor growth in vivo, suggesting other metabolites may compensate for the TCA cycle intermediates (such as glutamine) under MPC1 knockdown conditions. Pharmaceutical blocking MPC function induces a metabolic switch in PCa cells [56,57]. These results suggest that MPC plays an important role in tumor metabolic rewiring.

**PDK1–4**: The pyruvate dehydrogenase complex (PDC) is a gatekeeper controlling the flow of pyruvate toward the aerobic glycolysis or to mitochondria to produce acetyl-CoA to generate citrate for the TCA cycle. The activity of PDC is determined by pyruvate dehydrogenase kinase (PDK) and pyruvate dehydrogenase phosphatase (PDP). PDC is inactivated by PDK phosphorylation at its major subunit E1α (PDHA1) Ser293, Ser300 and Ser232 [58], and vice versa, it is activated by PDP de-phosphorylation [59].

**Figure 3:**
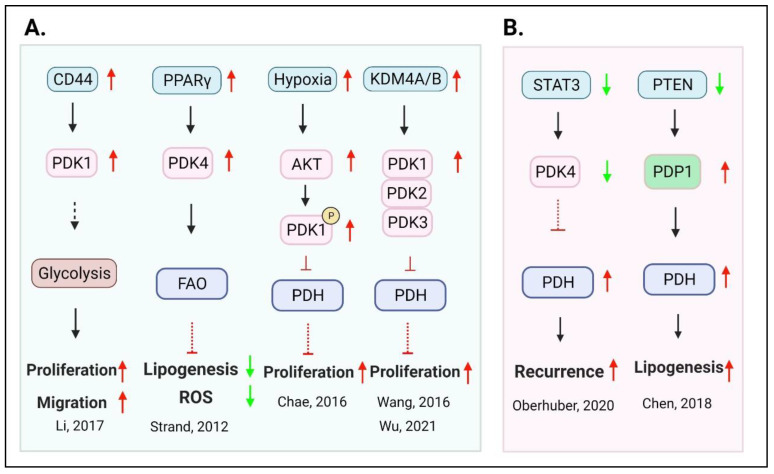
Duality of PDKs in PCa. (**A**) The high expression of PDKs regulated by CD44, PPARr, hypoxia, and KDM4A/B promotes PCa proliferation, migration in cell lines and xenograft models. (**B**) On the other hand, the low expression of PDK4 regulated by STAT3 leads to early recurrence and PCa lipogenesis by increasing PDH activity in clinical patient samples and transgenic PtenKO mouse model.

The four members of the PDK family (PDK1–4) are located in the mitochondrial matrix and share approximately 70% homology [60]. PDK1–3 are tissue-specific, and the expression of PDK4 depends on the energy state of the entire organism [61,62]. At high energy levels, PDKs can be activated by acetyl-CoA, ATP, and NADH. Conversely, the activity is inhibited by ADP, CoA, NAD+, and pyruvate at low energy levels [63]. Altered expressions of the PDK family members (PDK1–4) have been noticed in various types of malignant tumors, which are related to tumor proliferation, invasion, anti-apoptosis, and therapy resistance [64,65,66,67,68,69,70] (Figure 3). CD44, a transmembrane glycoprotein and stem cell marker, is commonly overexpressed in malignant tumor cells [71]. CD44 can regulate the glucose metabolism and ROS level in PCa cells, and its overexpression induced the level of PDK1, leading to cell proliferation and migration. Conversely, CD44 inhibitor SB-3CT decreases glucose consumption and increases the ROS levels in PCa cells [67].

Strand et al. demonstrated that overexpression of PPARγ in mouse prostate epithelial cells reduces lipogenesis, oxidative stress, and increased lactate secretion via the upregulation of PDK4 [72]. Consistently, knocking out of the major subunit of PDC complex, PDHA1, in PCa cells decreased the mitochondrial respiratory activity, enhanced migration ability, increased stemness, and poor prognosis [73]. PDK1 and PDK3 are directly transcriptionally upregulated by HIF1α, the hypoxia-inducing factor [74]. By activating PDK1, HIF-1a switches the metabolic pathway from mitochondria in favor of glycolysis to avoid damage to mouse embryo fibroblasts caused by excessive ROS production [75]. It was reported that in hypoxia, mitochondria Akt phosphorylates and activates PDK1 to sustain PCa cell proliferation [76]. Epigenetic regulators, such as KDM4A and B demethylases, which are overexpressed in PCa, also regulate PDK expressions. Thus, KDM4A, a histone demethylase, forms a complex with the E2F complex, which is recruited to the promoters of PDK1 and PDK3 to activate their transcriptions. KDM4A inhibition leads to overproduction of mitochondrial OXPHOS and ROS, resulting in cell death [77]. Likewise, KDM4B knockdown down-regulates the expressions of PDK1/2/3 and inhibits the proliferation of CRPC (C4-2B or CWR22RV1) [78]. The above in vitro studies suggest that enhanced PDK activities and reduced PDHA1 activities are important to maintain proper OXPHOS levels to sustain the growth and survival of PCa cells. Inhibition of PDK expression could lead to “overheating” of mitochondrial OXPHOS and ROS production, resulting in PCa cell death. A cautionary note is that cell cultures are often conducted in rich media where pyruvate flow to mitochondria is already robust; any further increase may not be tolerated. It also suggests that a balance of glycolysis vs. OXPHOS is so very critical in PCa cell growth.

By contrast, studies in the mouse (PtenKO) PCa model suggest that TCA cycle and OXPHOS activities are crucial for tumor progression [79,80]. Increased PDK activities are tumor suppressive [79], and PDC/PDHA1 activities are required for tumor growth [80]. In clinical specimens and the PtenKO model. Oberhuber et al. showed that a lower level of STAT3 in human primary prostate tumors is correlated with a higher expression of TCA and OXPHOS protein as well as a higher recurrence rate [79]. These observations were confirmed in PtenStat3 double knockout mice. The authors further showed that STAT3 activated PDK expression by directly binding to the PDK promoters. In both the clinical specimens and the PtenKO model, PDK expression was found to be downmodulated in parallel with STAT3. This study may reflect the difference between human cancer tissue and cancer cell lines [81]. Chen et al. studied the role of PDC in the Pten knockout model and found higher expression of PDP and PDC activity [80]. The double knockout of Pten and the major subunit of PDC, PdhA1, suppressed the tumor growth, which was accompanied by lower levels of TCA cycle activities and fatty acid synthesis. Glycolysis and lactate production, however, was not affected. In their systems, silencing PDHA1 suppressed OXPHOS and the PCa xenograft growth. These studies again underscore the importance of maintaining the proper level of OXPHOS and mitochondrial activities in PCa growth.

**LDH:** Lactate dehydrogenase (LDH) is an enzyme that catalyzes the conversion between pyruvate and lactate. As an increased influx of pyruvate into lactate was observed in higher grade and metastatic tissue, overexpression of lactate transporters (monocarboxylate transporters, MCTs) is also detected at different stages of PCa [82,83], suggesting the lactate could be used by PCa cells. Indeed, de Bari et al. identified a novel mitochondrial LDH in PCa cells, which can convert the mitochondrial lactate into pyruvate with increased mitochondrial activity in PCa [84,85]. These data suggest the lactate also can be another source for the pyruvate flux into the TCA cycle, leading to increased mitochondrial respiration activity (Figure 1).

**IDH:** Isocitrate dehydrogenase (IDH) is an enzyme that catalyzes the oxidative decarboxylation of isocitrate to produce α-ketoglutarate (α-KG) and CO_2_. Mutations in IDH1/2 cause loss of function in the normal TCA cycle and lead to the accumulation of the onco-metabolite 2-hydroxyglutarate (2HG), causing cancer such as acute leukemia and gliomas. Liu et al. propose that mammalian cells predominantly use the TCA cycle in the G1 phase but prefer glycolysis in the S phase. An elevated level of Skp2, a ubiquitin ligase, in PCa cells degrades IDH1 to favor glycolysis and replication in the S phase with consequent tumorigenesis. Conversely, adding non-degradable IDH1 restricts cell proliferation and xenograft tumor mass [86]. Wang’s study shows that silencing of IDH2 in prostate cancer cells increases glucose consumption and lactate production as well as the production of ROS and leads to the expression of the HIF1a pathway with increased tumor cell invasion but reduces the tumor cell proliferation and tumor volume [87].

**SIRTs:** Sirtuins (SIRTs) are NAD^+^-dependent histone deacetylases (HDACs) with a role involved in stress, inflammation, differentiation, and cancer. When activated, sirtuins could increase mitochondria activity, improve energy use efficiency, and reduce the damage of ROS. In PCa cells, SIRT1 located in nuclei controls the mitochondrial population by deacetylating PGC-1α and inhibiting mitophagy by the activation of SOD2 to reduce ROS [87]. Jaiswal showed that SIRT3 exhibits tumor suppressor activity in PCa. AR and steroid receptor coactivator 2 (SRC) activate HDAC2, which in turn inhibits the transcription of SIRT3, thereby increasing ACO_2_ activity and leading to mitochondrial citrate synthesis to promote prostate cancer growth [88]. Resveratrol, a polyphenol and known as a SIRT1 activator, could slow PCa cell growth by interfering with glycolysis and promoting respiration [89]. Selenium supplements, which also induce SIRT1 expression [90], can induce apoptosis by ROS production in PCa cells [91].

## 5. Genetic Regulation of OXPHOS in PCa

Besides the alteration of metabolic enzyme expression, the mitochondrial genome variation was extensively demonstrated in various types of cancer and considered as a potential biomarker for tumor progression [92,93,94,95]. In 2005, Petro et al. first observed the mitochondrial DNA (mt DNA) mutations in PCa [96]. They found that about 10% of PCa patient samples harbored mitochondrial genomic mutations in the coding sequence cytochrome oxidase subunit I (COI). The cybrid studies revealed that mtDNA mutation increases ROS production and tumor growth. Subsequently, Sun et al. also identified the mutation on COI and found this mutation is associated with the drug resistance in PCa [97]. Emerging data suggest that selected mtDNA mutation is associated with PCa risks, metastasis, and tumor recurrence [95]. Recently, Schöpf et al. discovered that there was a metabolic shift from glutamate/malate (complex I)-driven OXPHOS in benign tissue to succinate (complex II)-driven OXPHOS in high-grade PCa [98]. After mtDNA sequencing and profiling, they revealed that this shift is mainly caused by mtDNA mutation on complex I, leading to overexpression of complex II as well as a succinate-linked metabolic pathway. Importantly, this respiratory-shift phenotype is highly associated with survival. Consistently, Sant’Anna-Silva et al. showed that succinate accumulation increases the mitochondrial functions and malignancy in PCa cells [99]. As this is a tumor-specific event, targeting OXPHOS is a potential therapeutic strategy for PCa.

## 6. Targeting OXPHOS for PCa Therapy

### 6.1. Preclinical Studies

The studies reviewed above suggest balanced glycolysis and OXPHOS is important in maintaining the survival of tumor cells. Therefore, a potential therapeutic strategy for PCa is to disrupt such a balance via the manipulation of key regulators of this process, such as PDKs. Indeed, a number of PDK small molecule inhibitors were evaluated in preclinical studies [100]. The first used clinically as a PDK inhibitor is dichloroacetate (DCA) [101]. The efficacy of DCA in different cancer types has been extensively studied by several independent groups [102,103,104]. DCA suppresses the cancer cell growth by p53 induction and inhibition of HIF1α nuclear translocation, which leads to cell apoptosis [105]. Similar to DCA, melatonin reduces glycolytic activity by competing for the glucose uptake with GLUT1 in PCa [106,107]. Moreover, melatonin can be transported into mitochondria through the oligopeptide transporter, PEPT1/2. Huo et al. showed that accumulation of melatonin in mitochondria induces ROS production, which leads to caspase-dependent apoptosis in PCa cells [108]. In addition, melatonin shows an adverse effect on AR signaling by activating PKC, which induces the exit of AR from the nucleus [109]. The synergistic effect of melatonin with androgen deprivation therapies is currently under evaluation [109].

While metabolic balance is critical in PCa progression, a metabolic shift can confer drug resistance. Ippolito et al. showed that in docetaxel-resistant PCa cells, there was a suppression of the expression of aerobic glycolytic enzymes, such as MCT4, HK2, and TIGAR, and decreased glucose uptake due to downregulation of glucose transporter GLUT1 expression. At the same time, OXPHOS regulatory genes, such as PDHA1, MCT1, DLD, MYC, and PPARGC1A, are upregulated, suggesting a metabolic shift had occurred from glycolytic flux to OXPHOS in the acquisition of drug resistance [110]. This study implied that OXPHOS is not only important for early development but also for therapy resistance. Indeed, the authors showed that OXPHOS complex I inhibitor metformin reduced the survival in drug-resistance PCa cells [110]. In addition, Lee et al. showed that another complex I inhibitor, phenformin, effectively overcomes the drug resistance by blocking pro-survival autophagy in PCa cells and suppresses the tumor growth in a xenograft model [111]. Likewise, phenethyl isothiocyanate (PEITC), a complex III inhibitor, suppresses the OXPHOS function and activates ROS-dependent cell death in human PCa cells [112]. Many other compounds that can induce mitochondrial ROS production and disrupt mitochondrial functions are being evaluated in PCa treatment, such as methiothepin mesylate [113], triterpenoid [114], curcumin [115,116], jasmonate [117], levobupivacaine [118], and leucinostatin [119].

Recently, Zhang et al. showed that targeting a rate-limiting enzyme of fatty acid synthesis, ACACA, also affects mitochondrial bioenergetic profile [120]. Silencing ACACA reduces the mitochondrial respiration activity and mt copy number but increases the level of NAD+ and ROS, which leads to mitochondrial dysfunction in vitro. In vivo, silencing ACACA suppresses tumor growth. These results further reinforced the view that the OXPHOS pathway represents potential targets for PCa therapy.

### 6.2. Clinical Studies

Currently, five OXPHOS complex I inhibitors (metformin, phenformin, carboxyamidotriazole orotate, IACS-010750, and ME-344) are under clinical trials for cancer therapy [121]. Among these five OXPHOS inhibitors, metformin is the most extensively evaluated in many types of cancers, including breast cancer, oral cancer, and PCa, and it has reached phase IV clinical trials in oral cancer (NCT03684707).

For PCa, metformin is the only OXPHOS inhibitor evaluated in PCa patients with or without combined radio-, chemo- or androgen deprivation therapy (ADT) (Table 1). Over 50% of castration-resistant patients with monotherapy of metformin showed optimistic PSA response (Trial: NCT01243385). However, metformin did not show additional benefits for patients with combined radiotherapy and/or ADT (trial: NCT01620593, NCT01796028, NCT01677897), implying that metformin targets may overlap with radiotherapy (such as DNA synthesis [122]) or ADT targets as described previously. Moreover, metformin affects not only OXPHOS but also glucose and fatty acid metabolic pathways, likely to have a broader effect on radiotherapy or ADT [123]. Metformin is non-discriminatory between normal and tumor cells. In the future, targeting tumor-specific OXPHOS regulators may further improve the therapeutic efficacy.

## 7. Discussion

There is growing awareness that cancer progression is associated with metabolic abnormalities. Targeting key metabolic regulators such as OXPHOS has become a promising strategy for cancer therapy [121,133]. In this review, we discussed therapies that directly target OXPHOS components. There are, however, other metabolic inhibitors that indirectly affect OXPHOS. For instance, GLUT1 inhibitors (e.g., Ritonavir, Fasentin), hexokinase inhibitors (e.g., Ionidamine, 3-BrPA), PKM2 inhibitors (e.g., OA, TT-232, VK3, VK5), and PDK1 inhibitor (DCA) are metabolic inhibitors that have been evaluated in a variety of cancer types with Warburg phenotype [134]. Their clinical effects on PCa are currently unknown. Virtually all PCa carries deficiency in the arginine synthesis pathway, and arginine deprivation by ADI (arginine deiminase) was shown to be highly effective and selective in killing cancer cells. Phase 1 and 2 clinical trials for ADI-PEG20 in PCa have been completed in PCa [135]. ADI was shown to have a devastating effect on PCa OXPHOS and mitochondrial activities by mislocating TEAD4 [38,136]. PCa is also characterized by overexpression of FASN [137], and its inhibitor Omeprazole has gone through phase 2 (NCT04337580). Its impact on OXPHOS has yet to be determined. Additional metabolic inhibitors that affect OXPHOS in PCa are likely to be developed in the future.

The prostate has unique energy metabolism and special biology of producing and secreting citrate for seminal fluid. Unlike other cancers with Warburg phenotypes, the OXPHOS activities of PCa actually increase, as compared to normal prostate epithelial cells. This phenotype persists to advanced and drug-resistant stages [20]. As such, OXPHOS and mitochondrial activities play important roles in PCa progression, and its inhibition could lead to cell death. At the same time, as OXPHOS is already “loaded” in PCa cells, further increase; for instance, inhibiting PDKs and increasing pyruvate influx to mitochondria could generate excessive ROS and cause cell death. Thus, the OXPHOS pathway in PCa represents highly vulnerable targets for therapeutic interventions.

Most of the inhibitors target components of the OXPHOS fundamental processes, and an often-raised issue is that such metabolic inhibitors may also affect the mitochondrial functions of normal tissues. Perhaps, it would be more appropriate to target tumor-specific or selective OXPHOS regulators, such as overexpressed PDKs, MPC2, or TEAD4. In such a way, it would exploit the special prostate biology and affect the balance between glycolysis and OXPHOS in tumor cells but not normal counterparts.

## Figures and Tables

**Table 1:** Clinical trials for metformin in prostate cancer treatment.

Start Date	NCT Number	Conditions	Interventions	Phases	Status	Ref.
Dec.-2021	NCT02497638	Prostate Cancer	Metformin|Atorvastatin	Phase 2	Withdrawn	
Oct.-2021	NCT05036226	Prostate Cancer Recurrent	Hydroxychloroquine, Metformin, Sirolimus| Dasatanib|Nelfinavir	Phase 1 and 2	Not yet recruiting	
Jun.-2021	NCT04926155	Metastatic Prostate Cancer	Metformin	Phase 2	Not yet recruiting	
Jun.-2021	NCT04925063	Metastatic Prostate Cancer	Metformin	Phase 2	Not yet recruiting	
Nov.-2020	NCT04621669	Prostate Adenocarcinoma	SHR3680|digoxin|Rosuvastatin calcium|metformin	Phase 1	Not yet recruiting	
Nov.-2020	NCT04536805	Prostate Cancer	Metformin|Radiation	Phase 1 and 2	Recruiting	
Jul.-2018	NCT03031821	Prostate Cancer	Metformin	Phase 3	Recruiting	
Jul.-2018	NCT03465345	Prostate Cancer	Metformin|Oligomeric Procyanidin Complex	Phase 1	Withdrawn	
Oct.-2017	NCT02945813	Prostate Cancer	Metformin|Radiation	Phase 2	Active, not recruiting	
Jan.-2017	NCT03137186	Prostate Cancer	Metformin	Phase 2	Unknown status	
Dec.-2016	NCT02946996	Prostate Cancer	Metformin|Oligomeric Procyanidin Complex	Phase 2	Recruiting	
Jun.-2016	NCT02339168	Prostate Cancer	Metformin|Enzalutamide	Phase 1	Active, not recruiting	
Jun.-2016	NCT02640534	Prostate Cancer	Metformin|Enzalutamide	Phase 2	Active, not recruiting	
Dec.-2015	NCT02614859	Cancer of Prostate	Metformin|Bicalutamide	Phase 2	Active, not recruiting	
Jul.-2015	NCT02511665	Prostate Cancer	Metformin|Radiation	Phase 4	Unknown status	
Jun.-2015	NCT02420652	Prostate Adenocarcinoma	Metformin|Aspirin	Phase 2	Terminated	
May-2015	NCT02431676	Breast Cancer|Prostate Cancer|Lung Cancer|Colon Cancer|Melanoma of Skin|Endometrial Cancer|Liver Cancer|Pancreatic Cancer|Rectal Cancer|Kidney Cancer	Metformin|Self-control weight loss	Phase 2	Completed	[124,125]
Sep.-2014	NCT01996696	Prostatic Neoplasm	Metformin	Phase 2	Unknown status	
Jul.-2014	NCT02376166	Prostate Cancer	Metformin	Not Applicable	Completed	[126]
Jun.-2014	NCT02176161	Prostate Cancer Recurrent	Metformin	Phase 2	Recruiting	
Oct.-2013	NCT01864096	Prostate Cancer	Metformin	Phase 3	Recruiting	
Aug.-2013	NCT01677897	Metastatic Prostate Cancer	Metformin|Abiraterone	Phase 2	Completed	[127]
Jan.-2013	NCT01733836	Prostate Cancer	Metformin	Phase 2	Withdrawn	
Jan.-2013	NCT01796028	Prostatic Neoplasms	Metformin|Taxotere	Phase 2	Completed	[128]
Jan.-2013	NCT02778776	Prostate Benign Hyperplasia	Metformin|Agave inulin	Phase 3	Completed	
Jan.-2012	NCT01561482	Prostate Carcinoma	Metformin|Simvastatin	Phase 2	Withdrawn	
Nov.-2011	NCT01433913	Prostate Adenocarcinoma	Metformin	Phase 2	Completed	[129,130]
Jun.-2011	NCT01478308	Prostate Cancer	Metformin|Docetaxel|Prednisone	Phase 2	Withdrawn	
Apr.-2011	NCT01620593	Prostate Cancer	Metformin	Phase 2	Completed	[129]
Dec.-2010	NCT01243385	Prostate Cancer	Metformin	Phase 2	Completed	[131]
Sep.-2010	NCT01215032	Prostate Cancer	Metformin	Phase 2	Terminated	
Jun.-2009	NCT00881725	Prostate Cancer	Metformin	Phase 2	Terminated	
Jul.-2005	NCT00268476	Prostate Cancer	Celecoxib|Docetaxel|Prednisolone|ADT|Zoledronic Acid| Abiraterone|Enzalutamide|Metformin|Transdermal Oestradiol|Radiation	Phase 2 and 3	Recruiting	[132]
